# Toxicological Evaluation of a Potential Immunosensitizer for Use as a Mucosal Adjuvant—*Bacillus thuringiensis* Cry1Ac Spore-Crystals: A Possible Inverse Agonist that Deserves Further Investigation

**DOI:** 10.3390/toxins7124881

**Published:** 2015-12-09

**Authors:** Bélin Poletto Mezzomo, Ana Luisa Miranda-Vilela, Cesar Koppe Grisolia

**Affiliations:** 1 Department of Genetics and Morphology, Institute of Biological Sciences, University of Brasilia, Brasilia/DF 70.910-900, Brazil; belinpmezzomo@yahoo.com.br; 2 Faculty of Medicine, Faciplac, Campus Gama, SIGA Área Especial N° 02 Setor Leste—Gama/DF 72.460-000, Brazil

**Keywords:** *Bacillus thuringiensis*, δ-endotoxins, Cry1Ac toxin, biosafety, hematoxicicity, genotoxicity

## Abstract

In addition to their applicability as biopesticides, *Bacillus* *thuringiensis* (Bt) Cry1Ac spore-crystals are being researched in the immunology field for their potential as adjuvants in mucosal and parenteral immunizations. We aimed to investigate the hematotoxicity and genotoxicity of Bt spore-crystals genetically modified to express Cry1Ac individually, administered orally (p.o.) or with a single intraperitoneal (i.p.) injection 24 h before euthanasia, to simulate the routes of mucosal and parenteral immunizations in Swiss mice. Blood samples were used to perform hemogram, and bone marrow was used for the micronucleus test. Cry1Ac presented cytotoxic effects on erythroid lineage in both routes, being more severe in the i.p. route, which also showed genotoxic effects. The greater severity noted in this route, mainly at 6.75 mg/kg, as well as the intermediate effects at 13.5 mg/kg, and the very low hematotoxicity at 27 mg/kg, suggested a possible inverse agonism. The higher immunogenicity for the p.o. route, particularly at 27 mg/kg, suggested that at this dose, Cry 1Ac could potentially be used as a mucosal adjuvant (but not in parenteral immunizations, due to the genotoxic effects observed). This potential should be investigated further, including making an evaluation of the proposed inverse agonism and carrying out cytokine profiling.

## 1. Introduction

Delta-endotoxins (δ-endotoxins) from *Bacillus* *thuringiensis* (Bt) are parasporal crystalline protein inclusions synthesized during the sporulation phase of the bacillus [1,2]. These inclusions, also called crystal proteins (Cry) or Cry toxins, are toxic to insect larvae of various orders, and so they have been used enthusiastically in the biological control of agricultural pests [3]. Cry are part of a group known as pore-forming toxins (PFTs), characterized by presenting conformational changes that facilitate their insertion and translocation to the cell membrane of the host, typically transforming from soluble monomeric proteins to oligomers that form transmembrane channels [1,2,4,5].

Most of the Cry proteins (prototoxins) with high insecticidal potential have a long chain equivalent to 120–250 kDa molecular weight [6,7]. The three-dimensional structure of these δ-endotoxins consists of two regions, the carboxy-terminal portion (*C*-terminal), which is more conserved, and the amino terminus (*N*-terminus), which is often variable and associated with its toxic spectrum [3,7,8]. These proteins act similarly in the target host. Generally, endotoxins are released as protoxins by the bacteria and converted into their active form in the alkaline pH of the digestive tract of susceptible larvae, where they are cleaved by digestive enzymes, releasing a toxic fragment of about 60 kDa [7,8,9]. This is capable of binding to the receptors of the intestinal lumen, inducing cell lysis by forming ionic pores in the membrane and unleashing a series of symptoms that are fatal to the larvae [1,7].

The effectiveness of Cry in controlling these larvae accentuates commercial interest in these toxins. In this respect, Cry1Ac stands out as a powerful weapon against agricultural pests. Cry1Ac toxin is a family member of the Cry1A δ-endotoxins, and its toxicity is also associated with its *N*-terminus [10]. Cry1Ac, together with other Cry1A toxins, such as Cry1Aa and Cry1Ab, is recognized in several lepidopteran insect species by at least two specific receptors in the luminal membrane of midgut epithelial cells: a cadherin-like protein and a glycosyl-phosphatidylinositol (GPI)-anchored aminopeptidase N [7].

In addition to its applicability for the biological control of pests, the Cry1Ac prototoxin from Bt is being researched in the field of immunology for its potential use as a carrier and/or adjuvant in mucosal and parenteral immunizations [11,12,13,14,15]. Adjuvants (immune potentiators or immunomodulators) have been used for decades to improve the immune response to vaccine antigens. The incorporation of adjuvants in vaccine formulations is aimed at enhancing, accelerating and prolonging the specific immune response towards vaccine antigens [16].

Two potent mucous adjuvants have been tested for the preparation of vaccines: the cholera toxin (CT) from *Vibrio* *cholerae*, and the heat-labile toxin (LT) of *Escherichia* *coli*. However, both are products from bacteria that are pathogenic to humans and certain mammals, preventing their use [11,14]. Indeed, there are few substances capable of exerting a potent effect on mucous membranes that are also well tolerated by humans. The discovery of substances with these properties would benefit the management of infections and the design of vaccines. In this context, Cry1Ac prototoxin has been reported as having immunogenic and adjuvant actions that are as potent as cholera toxins [12]. Also, immunological studies for the control of malaria, a tropical disease with high mortality rates in affected regions, shows that Cry1Ac can be an important ally in *Plasmodium* control [17].

Therefore, the applicability of Cry1Ac proteins as potential tools in combating diseases in humans and other mammals further highlights the importance of studies directed to the biosafety of non-target organisms. This is mainly because although Cry toxins have been considered harmless to humans and other vertebrates [2,18], studies by our group have demonstrated that Bt spore-crystals caused hematologic disturbances for the erythroid and lymphoid lineages of Swiss mice [19,20,21], indicating that each spore-crystal endotoxin presents a characteristic profile of toxicity and might be investigated individually [21].

The aim of this study was therefore to investigate, in Swiss albino mice, the hematotoxicity and genotoxicity of Bt spore-crystals genetically modified to express Cry1Ac individually, administered orally or with a single intraperitoneal injection 24 h before euthanasia, to simulate the routes of mucosal and parenteral immunizations.

## 2. Results

### 2.1. Erythrogram (Table 1)

**Table 1:** Results of erythrogram of Swiss white mice treated with Bt toxin Cry1Ac administered 24 h before euthanasia, orally (*per os*, p.o.) or with a single intraperitoneal (i.p.) injection. Control mice received filtered water (negative control) or cyclophosphamide (CP—positive control) at 25 mg/Kg.

Treatment	RBC (×10^6^/μL)	HGB (g/dL)	HCT (%)	MCH (pg)	MCHC (g/dL)	MCV (fL)	RDW (%)
Filtered water, p.o.	7.53 ± 0.28	11.90 ± 0.44	30.38 ± 1.02	15.83 ± 0.09	39.12 ± 0.41	40.40 ± 0.40	17.83 ± 0.41 ^#^
CP 25 mg/Kg, p.o.	7.60 ± 0.12 ^#^	12.05 ± 0.26	30.57 ± 0.66	15.85 ± 0.16	39.45 ± 0.75	40.22 ± 0.46	16.80 ± 0.60
Cry 1Ac 6.75 mg/kg, p.o.	8.07 ± 0.13 ^○,#^	12.02 ± 0.18	30.23 ± 0.48	14.87 ± 0.10 **^,^^○^	39.77 ± 0.29	37.45 ± 0.27 **^,^^○,#^	13.78 ± 0.19 **^,^^○,#^
Cry 1Ac 13.5 mg/kg, p.o.	8.30 ± 0.19 ^○,#^	12.53 ± 0.25	31.12 ± 0.71	15.10 ± 0.11 **^,^^○,#^	40.30 ± 0.29	37.50 ± 0.26 **^,^^○,#^	13.57 ± 0.16 **^,^^○,#^
Cry 1Ac 27 mg/kg, p.o.	7.98 ± 0.12 ^○^	12.25 ± 0.24	30.42 ± 0.56	15.33 ± 0.20	40.28 ± 0.56	38.08 ± 0.22 **^,^^○,#^	16.93 ± 0.36 ^○,b^
*p*-values	0.029	0.570	0.918	0.002	0.374	0.000	0.000
Filtered water, i.p.	7.94 ± 0.14	12.32 ± 0.22	31.53 ± 0.63	15.35 ± 0.26	39.10 ± 0.31	39.25 ± 0.6	16.27 ± 0.44
CP 25 mg/Kg, i.p.	8.12 ± 0.12	12.63 ± 0.24	31.22 ± 0.43	15.57 ± 0.17	40.48 ± 0.22	38.42 ± 0.28	16.63 ± 0.30
Cry 1Ac 6.75 mg/kg, i.p.	7.32 ± 0.17 *^,^^○^	11.43 ± 0.19	30.13 ± 0.34	15.65 ± 0.37	37.93 ± 0.57 ^○^	41.30 ± 1.15	17.90 ± 0.44 *^,^^○^
Cry 1Ac 13.5 mg/kg, i.p.	7.53 ± 0.13 ^○^	12.05 ± 0.27	30.57 ± 0.97	16.02 ± 0.16	39.52 ± 0.66	40.57 ± 0.67	17.90 ± 0.50 *
Cry 1Ac 27 mg/kg, i.p.	7.50 ± 0.50	11.87 ± 0.74	30.73 ± 1.70	15.87 ± 0.35	38.55 ± 0.69	41.15 ± 0.89	16.80 ± 0.61
*p*-values	0.184	0.284	0.853	0.483	0.024	0.059	0.061
Total *p*-values	0.005	0.435	0.972	0.007	0.017	0.000	0.000

Data were expressed as mean ± SEM (standard error of mean). RBC = Red Blood Cells; HGB = Hemoglobin; HCT = Hematocrit; MCV = Mean corpuscular volume; MCH = Mean corpuscular hemoglobin; MCHC = Mean corpuscular hemoglobin concentration; RDW = Red cell distribution width (represents an indication of the amount of variation—anisocytosis—in cell size); g/dL = grams per deciliter; fl = fentoliters; pg = picograms. For the oral route and total group (total *p*-values), *p*-values of HGB, HCT, MCHC and MCV were generated by ANOVA, while *p*-values of the other parameters were generated by the Kruskal-Wallis test. For the i.p. route, all *p*-values were generated by ANOVA. Asterisks indicate significant differences at levels of *: *p* < 0.05 and **: *p* < 0.01 in the comparisons with the negative controls; the symbol “^○^” indicates these differences compared to the positive controls; the lower-case letter “^b^” indicate significant difference with the dose of 13.5 mg/Kg (dose-effect relationship) in the same route; and the symbol “^#^”, between the p.o. and i.p. routes for the same treatment.

For the red blood cells (RBC) count, hemoglobin (HGB) and hematocrit (HCT), none of the tested Cry1Ac doses promoted significant differences compared to the negative control in the oral (p.o.) route. However, in the intraperitoneal (i.p.) route, the dose of 6.75 mg/kg promoted a significant decrease in the RBC count, although this was still inside the reference values described for mice [22,23]. For the other hematimetric indices, the effects of the treatments were almost exclusive to the oral route, where the doses of 6.75 and 13.5 mg/kg promoted a significant reduction in the mean corpuscular hemoglobin (MHC), mean corpuscular volume (MCV), and red cell distribution width (RDW), while at 27 mg/kg this happened only with MCV. For MHC and MCV such reductions were below the reference values [22,23].

Compared to the positive control, in the p.o. route, all the Cry1Ac tested doses significantly raised the RBC count, while in the i.p. route, the doses of 6.75 and 13.5 mg/kg significantly reduced it. Again, for the other hematimetric indices, the effects of the treatments were almost exclusive to the oral route, with similar statistical results obtained with the comparisons with the negative control. The dose-effect relationship was observed only for RDW in the oral route, where the dose of 27 mg/kg significantly increased this value with respect to the dose of 13.5 mg/kg. Significant differences between the two routes were observed for the positive control when compared to Cry1Ac at the doses of 6.75 and 13.5 in the values of RBC and RDW; 13.5 in the value of MCH; and in all doses in the value of MCV.

### 2.2. Leukogram (Table 2)

With respect to the negative control, only the dose of Cry1Ac 27 mg/kg orally administered promoted a significant increase in the white blood cells (WBC) count, although this remained inside the reference values [22,23]. Compared to the positive control, significant differences were observed only for the i.p. route for lymphocytes and neutrophils + monocytes after treatment with Cry1Ac at 13.5 and 27 mg/kg.

Statistical differences were also shown between the two routes for the values of lymphocytes, after treatment with Cry1Ac at 27 mg/kg; neutrophils + monocytes, after treatment with CP (positive control) and Cry1Ac at 6.75 and 13.5 mg/kg; and eosinophils, after treatment with Cry1Ac at 6.75 mg/kg.

**Table 2:** Results of leukogram of Swiss white mice treated with Bt toxin Cry1Ac administered 24 h before euthanasia, orally (*per os*, p.o.) or with a single intraperitoneal (i.p.) injection. Control mice received filtered water (negative control) or cyclophosphamide (CP—positive control) at 25 mg/Kg.

Treatment	WBC (×10^3^/μL)	Lymphocytes (×10^3^/μL)	Neutrophils + Monocytes (×10^3^/μL)	Eosinophils (×10^3^/μL)
Filtered water, p.o.	4.63 ± 0.74	3.35 ± 0.55	1.22 ± 0.20	0.07 ± 0.05
CP 25 mg/Kg, p.o.	5.37 ± 0.77	3.73 ± 0.53	1.58 ± 0.34 ^#^	0.05 ± 0.03
Cry 1Ac 6.75 mg/kg, p.o.	5.22 ± 0.56	4.05 ± 0.47	1.15 ± 0.12 ^#^	0.02 ± 0.02 ^#^
Cry 1Ac 13.5 mg/kg, p.o.	5.07 ± 0.68	3.75 ± 0.48	1.23 ± 0.33 ^#^	0.08 ± 0.05
Cry 1Ac 27 mg/kg, p.o.	6.90 ± 0.89 *	4.25 ± 0.45 ^#^	2.52 ± 0.66	0.13 ± 0.10
*p*-values	0.458	0.655	0.643	0.816
Filtered water, i.p.	5.00 ± 1.33	3.83 ± 0.65	1.05 ± 0.89	0.12 ± 0.04
CP 25 mg/Kg, i.p.	5.05 ± 0.49	4.83 ± 0.49	0.10 ± 0.00	0.12 ± 0.02
Cry 1Ac 6.75 mg/kg, i.p.	6.82 ± 1.35	3.43 ± 0.57	3.20 ± 0.78 ^○^	0.18 ± 0.06
Cry 1Ac 13.5 mg/kg, i.p.	5.58 ± 0.59	2.75 ± 0.31 ^○^	2.75 ± 0.42 ^○^	0.08 ± 0.03
Cry 1Ac 27 mg/kg, i.p.	5.25 ± 0.86	2.47 ± 0.35 ^○^	2.58 ± 0.63 ^○^	0.20 ± 0.06
*p*-values	0.686	0.018	0.002	0.541
Total *p*-values	0.766	0.078	0.000	0.140

Data were expressed as mean ± SEM (standard error of mean). WBC = White Blood Cells. For the oral route and total group (total *p*-values), *p*-values of were generated by the Kruskal-Wallis test. For the i.p. route, *p*-values of WBC and lymphocytes were generated by ANOVA, while other *p*-values were generated by the Kruskal-Wallis test. Asterisks indicate significant differences at levels of * *p* < 0.05 in the comparisons with the negative controls; the symbol “^○^” indicates these differences compared to the positive controls; and the symbol “^#^”, between the p.o. and i.p. routes for the same treatment.

### 2.3. Plateletgram (Table 3)

For the oral route, CP significantly reduced the platelet (PLT) number compared to the negative control, while Cry1Ac at 6.75 and 13.5 mg/kg significantly decreased values of the mean platelet volume (MPV), platelet large cell ratio (P-LCR) and platelet distribution width (PDW). There were also significant differences between these Cry treatments with the positive control (CP), for PLT, MPV and PDW. For the i.p. route, compared to the negative control, although there were no significant differences in the PLT count, there were significant increases in the values of MPV, after treatments with CP and Cry1Ac at all doses; P-LCR after treatment with CP and Cry1Ac at 13.5 and 27 mg/kg; and PDW after treatment with Cry1Ac at all concentrations, although these treatments with Cry1Ac only differed from the positive control in the values of PDW.

Values of MPV and PDW also presented significant differences between the oral and intraperitoneal routes, mainly for Cry1Ac at 6.75 and 13.5 mg/Kg, while this difference appeared for P-LCR only at the dose of 13.5 mg/kg.

**Table 3:** Results of plateletgram of Swiss white mice treated with Bt toxin Cry1Ac administered 24h before euthanasia, orally (*per os*, p.o.) or with a single intraperitoneal (i.p.) injection. Control mice received filtered water (negative control) or cyclophosphamide (CP—positive control) at 25 mg/Kg.

Treatment	PLT (×10^3^/μL)	MPV (fl)	P-LCR (%)	PDW (fl)
Filtered water, p.o.	1219.00 ± 56.64	6.93 ± 0.10 ^#^	10.88 ± 0.81 ^#^	6.95 ± 0.11
CP 25 mg/Kg, p.o.	978.67 ± 93.57 *	7.10 ± 0.30	11.23 ± 2.33	7.25 ± 0.24
Cry 1Ac 6.75 mg/kg, p.o.	1312.17 ± 83.67 ^○^	6.30 ± 0.08 **^,^^○,#^	7.33 ± 0.60 **^,^^○^	6.57 ± 0.02 *^,^^○,#^
Cry 1Ac 13.5 mg/kg, p.o.	1338.00 ± 57.97 ^○^	6.30 ± 0.12 *^,^^○,#^	7.62 ± 0.78 *^,^^#^	6.55 ± 0.04 *^,^^○,a,#^
Cry 1Ac 27 mg/kg, p.o.	1224.67 ± 58.93 ^○^	6.90 ± 0.09 ^○^	9.63 ± 0.88	7.03 ± 0.10 ^b,^^#^
*p*-values	0.029	0.001	0.024	0.002
Filtered water, i.p.	1137.50 ± 95.72	6.58 ± 0.05	7.08 ± 0.65	7.00 ± 0.10
CP 25 mg/Kg, i.p.	1133.00 ± 69.15	6.90 ± 0.09 *	9.67 ± 0.48 *	7.05 ± 0.11
Cry 1Ac 6.75 mg/kg, i.p.	1181.05 ± 67.69	7.05 ± 0.12 *	8.68 ± 0.78	7.42 ± 0.11 *^,^^○^
Cry 1Ac 13.5 mg/kg, i.p.	1225.00 ± 74.21	7.30 ± 0.14 **	10.67 ± 1.18 *	7.62 ± 0.11 **^,^^○^
Cry 1Ac 27 mg/kg, i.p.	1000.33 ± 90.73	7.27 ± 0.14 **	11.23 ± 0.89 *	7.57 ± 0.08 **^,^^○^
*p*-values	0.378	0.004	0.015	0.000
Total *p*-values	0.066	0.000	0.004	0.000

Data were expressed as mean ± SEM (standard error of mean). PLT = platelets; MPV = mean platelet volume; P-LCR = platelet large cell ratio; PDW = platelet distribution width; fl = fentoliters. For the oral route and total group (total *p*-values), *p*-values of were generated by the Kruskal-Wallis test. For the i.p. route, *p*-values of PLT and PDW lymphocytes were generated by ANOVA, while other *p*-values were generated by the Kruskal-Wallis test. Asterisks indicate significant differences at levels of *: *p* < 0.05 and **: *p* < 0.01 in the comparisons with the negative controls; the symbol “^○^” indicates these differences compared to the positive controls; the lower-case letters “^a^” and “^b^” indicate, respectively, significant differences with the doses of 6.75 and 13.5 mg/Kg (dose-effect relationship) in the same route; and the symbol “^#^”, between the p.o. and i.p. routes for the same treatment.

### 2.4. Micronucleus (MN) Test (Table 4)

In the oral route, a significant difference with respect to the negative control was only observed for the treatment with Cry1Ac at 6.75 mg/kg, which significantly reduced the cell proliferation index (%PCE). However, for the i.p. route, all treatments (CP and Cry1Ac) promoted a significantly increased micronucleus (MN) number in both normochromatic erythrocytes (NCE) and polychromatic erythrocytes (PCE). In the i.p. route, Cry1Ac administration at all doses also promoted a significant decrease in the cell proliferation index (%PCE).

Compared to the positive control, significant differences appeared in the oral route for Cry1Ac at 6.75 and 13.5 mg/kg in the MN-NCE and %PCE. In the i.p. route, these differences were for all Cry doses in the MN-NCE, MN-PCE and %PCE.

Thus, there were statistical differences between the p.o. and i.p. routes for the treatments with CP and Cry1Ac at 6.75 and 13.5 mg/kg in the number of MN-NCE; and in all treatments for the number of MN-PCE. Furthermore, both the negative and positive controls showed significant differences between the two routes for the %PCE, presenting increased %PCE in the i.p. route compared to the p.o. route.

**Table 4:** Micronuclear evaluation of bone marrow cells from Swiss white mice treated with Bt toxin Cry 1Ac administered 24 h before euthanasia, orally (*per os*, p.o.) or with a single intraperitoneal (i.p.) injection. Control mice received filtered water (negative control) or cyclophosphamide (CP—positive control) at 25 mg/Kg.

Treatment	MN-NCE	Polychromatic Erythrocytes (PCE)	
		MN-PCE	Cellular Proliferation Index (%PCE)
Filtered water, p.o.	2.00 ± 1.44	2.50 ± 1.43	52.61 ± 1.01 ^#^
CP 25 mg/Kg, p.o.	2.17 ± 0.48 ^#^	3.50 ± 0.62 ^#^	45.97 ± 1.21 *^,^^#^
Cry 1Ac 6.75 mg/kg, p.o.	0.83 ± 0.31 ^○,#^	2.33 ± 0.67 ^#^	53.08 ± 1.54 ^○^
Cry 1Ac 13.5 mg/kg, p.o.	0.83 ± 0.31 ^○,#^	2.00 ± 0.37 ^#^	52.38 ± 0.80 ^○^
Cry 1Ac 27 mg/kg, p.o.	1.33 ± 0.21	3.33 ± 0.76 ^#^	49.49 ± 2.14
*p*-values	0.179	0.307	0.007
Filtered water, i.p.	0.17 ± 0.17	2.33 ± 0.42	70.13 ± 0.87
CP 25 mg/Kg, i.p.	14.33 ± 1.99 **	26.83 ± 2.52 **	66.56 ± 1.30
Cry 1Ac 6.75 mg/kg, i.p.	4.17 ± 0.48 **^,^^○^	7.67 ± 1.23 **^,^^○^	47.99 ± 1.20 **^,^^○^
Cry 1Ac 13.5 mg/kg, i.p.	4.67 ± 1.15 **^,^^○^	6.00 ± 0.37 **^,^^○^	51.72 ± 0.95 **^,^^○^
Cry 1Ac 27 mg/kg, i.p.	2.50 ± 0.56 **^,^^○^	5.67 ± 0.56 **^,^^○^	51.36 ± 1.52 **^,^^○^
*p*-values	0.000	0.000	0.000
Total *p*-values	0.000	0.000	0.000

Data were expressed as mean ± SEM (standard error of mean). MN-NCE and MN-PCE = micronucleus results for normochromatic erythrocytes (NCE) and polychromatic erythrocytes (PCE), respectively. For the oral route and total group (total *p*-values), *p*-values of MN-NCE and MN-PCE were generated by the Kruskal-Wallis test; while *p*-value of %PCE was generated by ANOVA. For the i.p. route, *p*-values of MN-EPC and %EPC were generated by ANOVA, while *p*-value of MN-ENC was generated by the Kruskal-Wallis test. Asterisks indicate significant differences at levels of *: *p* < 0.05 and **: *p* < 0.01 in the comparisons with the negative controls; the symbol “^○^” indicates these differences compared to the positive controls; and the symbol “^#^”, between the p.o. and i.p. routes for the same treatment.

## 3. Discussion

The mechanism of action of the Cry proteins depends on their affinity for specific target receptors and their activation by proteolytic digestion in the alkaline pH of the digestive tract of their target organisms [1,7,24]. So, because there have been no reports of known receptors in the gut cells of the vertebrates and the physiology of their digestive system, δ-endotoxins have been considered as presenting inconsiderable toxicity to humans and other mammals [25,26]. However, the literature addressing the biosafety of δ-endotoxins for non-target organisms is scarce, and our present and previous works [19,21] have demonstrated hematotoxicity mainly for the erythroid lineage of mice after a single oral administration, corroborating another study demonstrating *in vitro* hemolysis in cell lines of rat, mouse, sheep, horse, and human erythrocytes [27]. This data suggests that the plasma membrane of erythrocytes could be the primary target for these toxins [19,27], and that Cry toxins are not completely destroyed in the stomach, having their toxic form activated by intestinal alkaline pH. However, some studies have shown that commercial Bt products are safe, and that the mortality observed in tests with mice was verified for extremely high doses and shown to be dependent on the route of administration [25,28], corroborating our previous report [20].

It has been reported that strains of Cry toxins can be solubilized by an alkaline buffer or a combination of an alkaline buffer and reducing conditions [27]. In our study, lyophilized Bt Cry1Ac spore-crystals resuspended in distilled water presented cytotoxic effects on erythroid lineage in both routes, p.o. and i.p. In the p.o. route, according to the results of erythrogram, Cry1Ac at 6.75 and 13.5 mg/Kg produced microcytosis (decreased MCV), with MCH decreasing proportionally with MCV below the reference values. In the i.p. route, despite the non-significant decreased MCHC and increased MCVvalues, the significant results for decreased RBC (in the erythrogram) and cellular proliferation index (%PCE in the MN test), as well as the increased RDW for Cry1Ac at 6.75 mg/Kg, indicate a greater severity for the systemic route mainly for this dose, possibly due to an inverse agonism [29].

According to the conventional two-state drug receptor interaction model, binding of a ligand may initiate activity (agonist with varying degrees of positive intrinsic activity) or prevent the effect of an agonist (antagonist with zero intrinsic activity). Inverse agonists bind with the constitutively active receptors, stabilize them in the inactive state, and thus reduce the activity (negative intrinsic activity) [29]. This could explain the more severe effects of Cry1Ac at 6.75 mg/kg, intermediate effects at 13.5 mg/kg, and milder effects at 27 mg/kg, because the latter could lead to a greater stabilization of the receptor in its inactive form than at 6.75 or 13.5 mg/kg. However, this suggestion deserves further investigation, particularly in the *in vitro* systems. This is because under physiological conditions, constitutive activity is usually low in natural receptors, since the fraction of unoccupied receptors in the active conformational state is minimal. Nonetheless, when the population of constitutive receptors is overexpressed in the *in vitro* systems, such as when expressed in high amounts in cultured cells, they exhibit significant and measurable spontaneous activity [29].

Outcomes also suggest that an early release of immature red blood cells could be occurring, a normal bone marrow response after hemolysis [23]. Because reticulocytes are larger and contain less hemoglobin than mature red cells, MCV is increased and MCHC is decreased, and because the presence of reticulocytes means that red cell size is more variable, anisocytosis (evaluated by RDW) is increased [22]. The severity of the i.p. route for the erythroid lineage was also demonstrated in the results of the MN test.

Mutagenicity testing is used to assess submicroscopic changes in the base sequence of DNA, chromosomal aberrations, and structural aberrations in DNA [30]. In this context, Cry1Ac administered by i.p. route showed a genotoxic effect at all doses, by inducing MN in both normochromatic and polychromatic erythrocytes. Decreased red cell life span implies that hemolysis is occurring at a faster than normal rate. Because the life span of mouse red blood cells is much shorter than in larger animals, the effect of hemolysis on red cell mass occurs more quickly [22]. It has been suggested that the frequency of MN in peripheral blood normochromatic erythrocytes is dose and duration dependent, while the decline in MN frequency after the end of exposure can be related to changes in the kinetics of erythropoiesis [31]. Although our work was not also carried out with MN test in peripheral blood leukocytes, results reinforce the need for more research to avoid the potential risks of the increased bioavailability of these microbiological control agents (MCA), especially given that little is known about spore crystals’ adverse effects on non-target species [19,20,26,32].

In mice, the inflammatory response is often associated with both increased lymphocytes and neutrophils, where small changes in the number of neutrophils may be biologically significant and reflected in the total leukocyte count [22]. In this context, results indicate higher immunogenicity for the p.o. route, particularly for the dose of 27 mg/kg. Although no cytokine profiling was performed in our study, this is an interesting result because, according to what is discussed above and in our previous reports [19,20], in this route and at this concentration there was no genotoxic effect and practically no or very few hematotoxic effects. This suggests that at 27 mg/kg, Cry 1Ac could have a potential use as a carrier and/or adjuvant in mucosal immunizations (but not in parenteral immunizations, due to the genotoxic effects observed). Results of plateletgram corroborate this.

## 4. Experimental Section

### 4.1. Chemicals

Ketamine chloridrate, sold as Dopalen 100 mg/mL, was obtained from Ceva Animal Health Ltda (São Paulo, Brazil); xylazine chloridrate (Coopazine^®^ 20 mg/mL) came from Coopers (São Paulo, Brazil).

### 4.2. Bt Cry1Ac Spore-Crystals

Bt spore-crystals from a Bt strain genetically modified to express Cry1Ac individually were obtained in lyophilized form from the Germplasm Bank of the Brazilian Agricultural Research Corporation (Embrapa, Brazil) through its National Genetic Resource and Biotechnology Research Center (Cenargen, Brasilia/DF, Brazil), as previously reported [19,20].

### 4.3. Animals and Experimental Design

Sixty Swiss albino mice of both genders, 10–12 weeks old, weighing 30.2 ± 2.2 g, were obtained from the Central Animal Facility of the University of São Paulo (Ribeirão Preto/SP, Brazil). After the period of acclimatization, a sample size of 6 mice, 50% male and 50% female, was used for each experimental group. They were randomly housed in plastic cages (3 of each gender/cage) under standard conditions at 22 ± 2 °C in a 12 h light/dark cycle and fed with standard diet and water *ad libitum*, as previously described [19,20].

Lyophilized Bt spore-crystal Cry1Ac was resuspended in distilled water at 37 °C, agitated for 10 min. and administered orally (*per os*, p.o.) [19] or intraperitoneally (i.p.) [20] with a single dose, 24 h before euthanasia, at concentrations of 6.75, 13.5 and 27 mg/kg. These concentrations were chosen from a pilot study previously carried out with four Bt spore-crystals, which showed that, for Cry1Ac, 27 mg/kg was the maximum dose, which could be administered by the i.p. route without deaths recorded (maximum tolerated dose) [20]. So, the lower exposures levels of 6.75 and 13.5 mg/kg were based on this maximum tolerated dose, and used to compare with it. Control groups received filtered water (negative controls) or cyclophosphamide at 25 mg/kg (CP, positive controls).

Animals were then anesthetized by an intraperitoneal administration of ketamine (80 mg/kg) plus xylazine (10 mg/kg), and blood samples collected by cardiac puncture (400 μL) were used to carry out hemogram in microtubes containing EDTA as anticoagulant, using a multiple automated hematologic analyzer for veterinary use, Sysmex pocH-100iV Diff (Curitiba/Paraná, Brazil) calibrated for mice. After that, animals were euthanized by cervical dislocation [22], bone marrow cells were surgically removed, the slides for the micronucleus (MN) test were prepared [23] and the genotoxic potential of Bt spore-crystals was evaluated [19].

All procedures were reviewed and approved by the institutional Ethics Committee for Animal Research (Institute of Biological Science, University of Brasília), number 32942/2009. Due to the requirement of the institutional Ethics Committee for Animal Research to reduce the number of animals used, the control groups (negative and positive) and the group treated with Cry 1Ac at 27 mg/kg used here also belonged to the previously published studies [19,20], which were performed at the same time.

### 4.4. Statistical Analysis

Statistical analysis was carried out using SPSS (Statistical Package for the Social Sciences) for Windows, version 17.0 (IBM SPSS Statistics). Data were expressed as mean ± SEM (standard error of mean) and values of *p* < 0.05 were considered statistically significant. The continuous variables were tested for normal distribution with Shapiro-Wilk. Possible differences among the analyzed groups were investigated through ANOVA or Kruskal-Wallis test (data not normally distributed), followed respectively by the Bonferroni or the Mann-Whitney U tests. Additionally, *p*-values with statistical significance (*p* < 0.05) were only considered when they also presented biological significance, according to the following criteria: (1) controls compared to all groups; (2) different doses in the same route (dose-effect relationship); and (3) the same dose and treatment in both the p.o. and i.p. routes.

## 5. Conclusions

In conclusion, Cry1Ac presented cytotoxic effects on erythroid lineage in both routes, being more severe in the i.p. route, which also showed genotoxic effects. The greater severity found for this systemic route, mainly at 6.75 mg/kg, the intermediary effects at 13.5 mg/kg, and practically no or very little hematotoxicity at 27 mg/kg suggested a possible inverse agonist. The higher immunogenicity for the p.o. route, particularly for the dose of 27 mg/kg, suggested that at this dose Cry 1Ac could have potential use as a carrier and/or adjuvant in mucosal immunizations (but not in parenteral immunizations, due to the genotoxic effects observed). However, this potential deserves further investigation, particularly in the *in vitro* systems, in which evaluations of the proposed inverse agonist and cytokine profiling should be performed.
